# Micropropagation of transgenic lettuce containing HBsAg as a method of mass-scale production of standardised plant material for biofarming purposes

**DOI:** 10.1007/s00299-016-2056-1

**Published:** 2016-09-21

**Authors:** Tomasz Pniewski, Marcin Czyż, Katarzyna Wyrwa, Piotr Bociąg, Paweł Krajewski, Józef Kapusta

**Affiliations:** 1 Institute of Plant Genetics, Polish Academy of Sciences, Strzeszyńska 34, 60-479 Poznań, Poland; 2 Institute of Biotechnology and Antibiotics, Starościńska 5, 05-216 Warsaw, Poland

**Keywords:** HBV surface antigens, HBsAg, Micropropagation, Lettuce, Oral vaccine, Plant material standardisation

## Abstract

*****Key message***:**

**Micropropagation protocol of transgenic lettuce bearing S-, M- and L-HBsAg was developed for increased production of uniformised material for oral vaccine preparation.**

**Abstract:**

Effective manufacturing of plant-based biopharmaceuticals, including oral vaccines, depends on sufficient content of a protein of interest in the initial material and its efficient conversion into an administrable formulation. However, stable production of plants with a uniformised antigen content is equally important for reproducible processing. This can be provided by micropropagation techniques. Here, we present a protocol for micropropagation of transgenic lettuce lines bearing HBV surface antigens: S-, M- and L-HBsAg. These were multiplied through axillary buds to avoid the risk of somaclonal variation. Micropropagation effectiveness reached 3.5–5.7 per passage, which implies potential production of up to 6600 plant clones within a maximum 5 months. Multiplication and rooting rates were statistically homogenous for most transgenic and control plants. For most lines, more than 90 % of clones obtained via in vitro micropropagation had HBsAg content as high as reference plants directly developed from seeds. Clones were also several times more uniform in HBsAg expression. Variation coefficients of HBsAg content did not exceed 10 % for approximately 40–85 % of clones, or reached a maximum 20 % for 90 % of all clones. Tissue culture did not affect total and leaf biomass yields. Seed production for clones was decreased insignificantly and did not impact progeny condition. Micropropagation facilitates a substantial increase in the production of lettuce plants with high and considerably equalised HBsAg contents. This, together with the previously reported optimisation of plant tissue processing and its long-term stability, constitutes a successive step in manufacturing of a standardised anti-HBV oral vaccine of reliable efficacy.

## Introduction

Plants are becoming more and more important biofactories, providing vaccines, antibodies and other biopharmaceuticals. Some of them have been already licensed, such as the enzyme glucocerebrosidase (taliglucerase alfa, Protalix™) against Gaucher’s disease, or are very close to implementation, such as a vaccine against influenza (haemagglutinins assembled into virus-like particles, Medicago Inc.) (Grabowski et al. 2014; Ward et al. 2014). Nowadays, most of biopharmaceuticals are produced using transient expression systems based on virus-derived vectors or agroinfiltration (Thuenemann et al. 2013; Yusibov et al. 2013). Despite the unquestionable effectiveness of these systems, biopharmaceuticals produced in this manner usually require to be purified. Therefore, these are aimed mostly as parenterally delivered preparations rather than oral delivery.

The original idea of low cost and commonly available oral vaccines founded on transgenic plants producing antigens dates back more than 20 years. Among oral vaccines, the one against HBV (Hepatitis B Virus) has been a key example. During the past decades, the concept of an anti-HBV vaccine evolved from raw tissues used as ‘edible’ vaccines to orally administered derivatives obtained from converted plant tissue (Pniewski 2014). Recently, great progress has been made in the expression of all HBV surface antigens: small S-, medium M- and large L-HBsAg in donor plants, as well as in technologies for their conversion into immunogenic, highly condensed and durable formulations. In the case of S-HBsAg, the expression yield reached around 60 or 70 µg/g FW in lettuce or maize, respectively, or 16–17 µg/g FW for M- and L-HBsAg in lettuce (Hayden et al. 2012a; Pniewski et al. 2011, 2012). Maize grains were treated with organic solvents to remove fats and the obtained pulp was pelleted or wafered (Hayden et al. 2012b, 2014), while lettuce leaves were processed into lyophilisate (Czyż et al. 2014, 2016). Lyophilised lettuce tissue was also used as a vehicle of an oral vaccine against tuberculosis and to induce tolerance in haemophilia therapy (Lakshmi et al. 2013; Su et al. 2015). Lettuce was chosen as a producer of oral vaccines since it is a plant whose leaves are consumed raw and is free of harmful substances, and thus suitable for preparation of derived oral formulations. Moreover, it can be cultivated in various conditions: greenhouse, foil tunnels, in the field and in hydroponic systems. However, high antigen content and efficiency of plant material processing are not the only necessary conditions required to produce potential oral medicines. It should be equally important to ensure a stable antigen expression and/or uniformised and repeatable production of initial material to be processed as it is for pharmaceuticals from herbal and medicinal plants (Parveen et al. 2015; Engisch and Muzzio 2016).

Generation of plants stably producing orally administered biopharmaceuticals is still a challenge. Although for some of them it has been possible to obtain transplastomic plants (Lakshmi et al. 2013; Su et al. 2015), most antigens designed as oral vaccines are still produced in transgenic plants. However, the activity of a transgene is affected by a complex interaction with the host genome, involving position and gene dosage effects, gene silencing and other processes (Gelvin and Kim 2007; Miki et al. 2009). Random integration of a transgene results in a pool of new plant transgenic genotypes, expressing various phenotypes. A desired phenotype should then be selected and propagated with a simultaneous preservation of valuable traits. This can be achieved with the use of breeding methods such as backcrossing and inbreeding. In the case of maize expressing S-HBsAg, such operations assured not only stabilisation, but also a tenfold increase of the antigen production (Hayden et al. 2015). Albeit effective, these classical methods require several breeding cycles followed by thorough analyses.

Alternatively, pre-selected plant lines may be proliferated by vegetative propagation, which enables the mass production of clones of the same unaltered genotype. This method exploits the innate ability of many wild and crop plant species to propagate asexually by special organs (rhizomes, stolons, miniaturised plantlets, etc.) or even any fragments containing meristematic cells. For several years, specialised methods of vegetative propagation, including cell and tissue cultures, were developed for many utilitarian species (Bajaj 1991, 1992, 1997; Debergh and Zimmerman 1991). In vitro cultures, apart from the use in transformation procedures, are also considered as one of the basic tools for generation and production of unconventional plant lines (Loberant and Altman 2010).

Here, we present clonal micropropagation of transgenic lettuce expressing S-, M- and L-HBsAg, as a system enabling mass production of uniformised plant material for an oral vaccine preparation with a potential use for other biopharmaceuticals.

## Materials and methods

### Plant material

To establish the basic conditions of micropropagation, a pilot experiment with the use of non-transgenic lettuce (*Lactuca sativa*) cv. Syrena was performed, where the type of initial explants from germinated seedling (with or without cotyledons) and media sequence (see next section) were tested. In the main micropropagation experiments, plants of the T1 generation—progeny of previously obtained parental transgenic lettuce lines (LT)—were used. The lines were grouped according to the expressed HBs antigen: LT10—S-HBsAg, LT9A—M-HBsAg and LT11—L-HBsAg (Pniewski et al. 2011, 2012). Within each group, three lines were chosen, varying in their HBsAg expression levels in the T0 generation and/or the number of transgene integration sites (abbreviated as ‘TIS’): for LT10—4D, 6A and 26G (9.9, 10.9, 45.4 µg/g FW and 1, 2, 1 TIS, respectively); LT9A—1E, 15E and 18A (4.7, 10.5, 4.2 µg/g FW and 1, 2, 2 TIS, respectively); LT11—6D, 17A and 18C (13.3, 7.6, 4.1 µg/g FW and 3, 2, 1 TIS, respectively). These lines were selected on the basis of primary tests for HBsAg expression in ten plants of the T1 generation, where the expression was similar to that for T0 plants. Each chosen line as well as the non-transgenic control was divided into micropropagated and reference plants. The latter were obtained directly from seeds, developed without a period of in vitro culture and grown constantly under standard greenhouse conditions.

### In vitro micropropagation course

Seeds, 30–35 for a line, were sterilised for 12 min with 20 % v/v chloric bleach supplemented with 0.01 % v/v Tween^®^20, washed five to six times with distilled water and germinated (passage *n*-0) in the semi-shade at 25 °C for 3 days on 0.8 % w/v agar (Serva). In vitro cultures were conducted under 4000 lx of light intensity (T8 Fluora lamps), 16/8 h photoperiod and at a 25/18 °C temperature regime. All media used for multiplication consisted of SH salts (Schenk and Hildebrandt 1972) and B5 vitamins (Gamborg et al. 1968), but with an increased inositol content (to 1 g/L), and were supplemented with sucrose (30 g/L) and KIN (concentration depending on the culture step). Media were adjusted to pH 5.7, solidified with agar (0.8 % w/v) and supplemented with glufosinate ammonium (2.5 mg/L) for cultures of LT plantlets. After germination, roots and cotyledons were removed and such truncated initial seedlings were transferred onto Petri dishes with the axillary bud induction medium LM1, supplemented with KIN (5 mg/L). At this step developing plantlets were verified for transgene presence using PCR as described previously (Pniewski et al. 2011, 2012). PCR-positive initial seedlings were cultured on LM1 medium for two 10-day passages (*n*-1 and *n*-2). Then developed multiplantlets were split for the first time and derived (multi)plantlets were transferred onto LM2 medium, with a reduced KIN content (0.5 mg/L). Culture on LM2 was conducted for five 3-week passages (*n*-3 to *n*-7). During each transfer, random multiplantlets were divided and derivatives were counted. For culture continuation, a maximum of 15 derivatives for an initial seedling were retained, while a maximum of 3 were transferred to jars for rooting. The rooting medium consisted of half a dose of SH macroelements, a full dose of SH microelements, B5 vitamins and sucrose (30 g/L). Plants which rooted within 3–4 weeks were transferred into soil and grown ex vitro in a greenhouse.

### Plant cultivation in greenhouse and analyses

Seeds for reference plants were germinated with about a 10-week delay to develop plants at approximately the same time as the first expected ex vitro clones. Seeds were sterilised as above and germinated under the same conditions on filter paper. Seedlings were transferred to multiplates with soil and then PCR verified for a transgene presence as above. PCR-positive reference plants as well as successively obtained ex vitro clones were cultivated in pots in a greenhouse under 15–20 klx light intensity, 16/8 h photoperiod and at a 22/16 °C temperature regime. Both plant types were analysed for S-, M- and L-HBsAg contents using ELISA four-repetition tests with antibodies specific to the common S domain, as described previously (Pniewski et al. 2011, 2012). HBsAg content was assayed two times during plant growth—at the stage of fully formed heads and at the very beginning of the formation of inflorescence stems. Also at the latter stage, leaf and total biomass of randomly selected plants was determined. The remaining plants were cultivated till seed development.

### Calculations and statistical analysis

Multiplication efficiency was calculated as a ratio of the obtained plants (maximum 15 per division round) to the used ones. This parameter was calculated for each initial plant (seedling) of a given line and then as an arithmetic mean together with SEM for the whole line in an individual passage. The general multiplication efficiency was calculated as a geometric mean. Rooting efficiency was expressed as an arithmetic mean together with SEM of successfully rooted plants. Micropropagation effectiveness was calculated as a mean number of clones obtained in a single passage from a single initial plant, i.e. the product of general multiplication and rooting coefficient. The mean leaf or total biomass—10 for the reference plants and 30 for clones (in this case, 6 per each of 5 multiplication passages) and the number of seeds (for all remaining plants) for particular lines were calculated as arithmetic means together with SEM. Multiplication efficiencies in individual passages within a line or among lines and general ones for all passages (geometric means after log(*x*) transformation), as well as rooting efficiency and plant growth parameters were compared using one-way ANOVA with the post hoc Tukey test at *p* = 0.01, using the Statistica 8.0 statistical software package (StatSoft Inc, USA).

The mean HBsAg expression values were calculated from two measurements for each clone of an initial plant (seedling) or a reference plant within a line. The number of analysed clones amounted from six (exceptionally 4) to eight per an initial plant. These data were then analysed statistically (after log_2_(*x*) transformation, for each plant line separately) and compared for clones vs. reference plants by analysis of variance in a mixed model involving fixed effects of propagated (i.e. cloned) plants and random effects of clones and allowing for heterogeneous variance between clones within initial plants, using the Genstat Edition 17 package (VSN Int.).

## Results

### Micropropagation

**Fig. 1:**
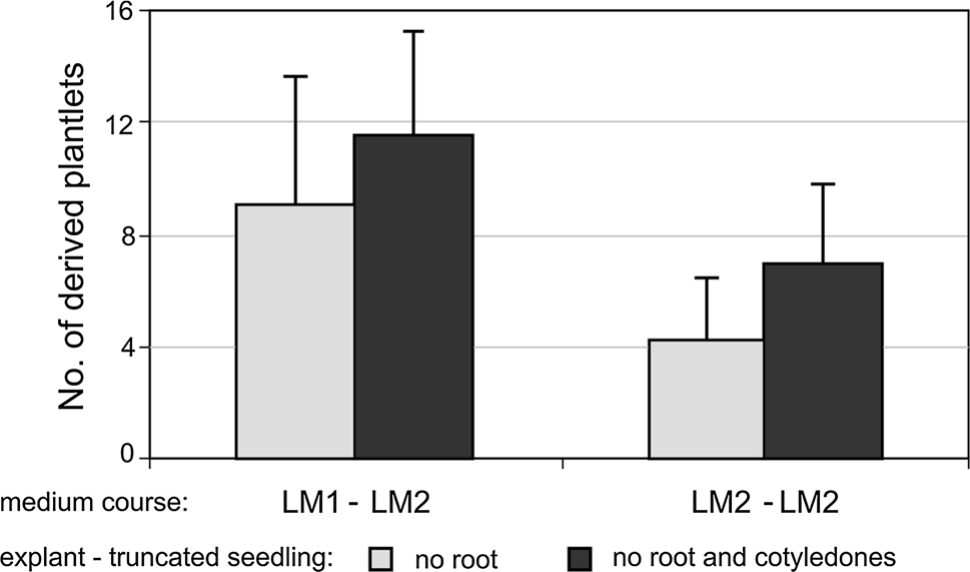
The effect of initial explant and medium course on efficiency of in vitro lettuce multiplication

In the pilot experiment, initial conditions of in vitro culture steps were established. The factors evaluated included the type of the initial explant from the germinated seedling: I—with the excised root only; or II—excised root and cotyledons (35 of each); and media sequence: LM1-LM2 or LM2-LM2. The protocols of in vitro culture analysed did not differ significantly in effectiveness regarding the numbers of plantlets derived (not shown). Yet, the combination of explants type I and LM1–LM2 transfer was on average 1.3–2.7 times more effective than the others (Fig. 1). Hence, this protocol was adopted in the main part of the research concerning the micropropagation of transgenic lettuce lines.

**Fig. 2:**
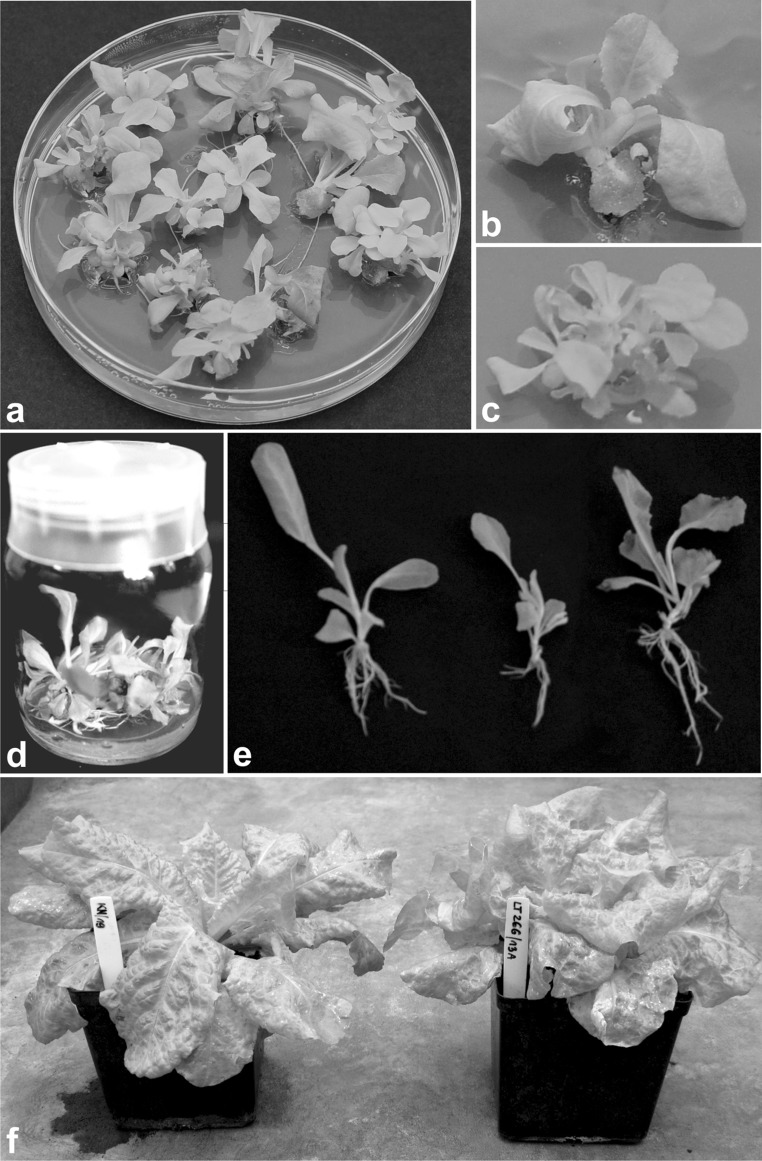
The course of lettuce micropropagation: **a** plantlet multiplication in vitro; **b** early stage of formation of sequent plantlets from stimulated axillary buds of an initial plantlet; **c** fully developed multiplantlet before fission; **d** later stage of plantlet growth and rooting in vitro; **e** rooted plants to transfer to soil; **f** ex vitro growth of transgenic lettuce plant (on the *right*) in comparison to non-transgenic control constantly growing in greenhouse (on the *left*)

Most (70–100 %) developed seedlings—progeny of primary transformants—were PCR positive regarding transgene presence (data not shown). These were grown in a greenhouse as reference plants or used for the micropropagation experiment. During this process (Fig. 2), new plantlets developed from axillary buds, stimulated by KIN (Fig. 2a–c). Induction of plantlet formation from initial seedlings, but still without fission, was performed on LM1 medium. Multiplantlets (Fig. 2b, c) were then moved onto LM2 medium and cultured for five passages with accompanying division. During each transfer, any developed callus was removed and multiplantlets were usually split into several smaller ones—to obtain a maximum of 15 derivatives for an initial seedling. Randomly derived plantlets were excised as individuals and transferred onto the rooting medium (Fig. 2d). Micropropagation was performed till six to eight (or, exceptionally, 4 for line LT11-18C) clones for an initial plant (seedling), or a minimum of 100 clones per whole line were successfully rooted and adapted to ex vitro conditions (Fig. 2e, f).

Individual lines of transgenic lettuce varied in multiplication efficiency across the course of micropropagation passages (Table 1; analyses of particular lines are shown across each row in the grey-coloured part, with distinct groups denoted by lowercase letters). Some of them formed new plantlets quite uniformly, for example lines LT10-26G and LT9A-1E which exhibited no significant changes (one statistical group) throughout micropropagation, while for others multiplication fluctuated considerably between passages. Multiplication of LT lettuce in individual passages did not differ from the control, except for some rare cases (Table 1, analysis for particular passages shown within columns in the grey-coloured part, with significant differences marked by asterisks). Nonetheless, general multiplication effectiveness (4.5–6.0) did not differ significantly among most of the lines, while none of the LT lines differed (Table 1, analysis in ‘multiplication’ column, marked by upper case letters) from non-transgenic control lettuce (5.6). Similarly, clones of most transgenic lines and control lettuce rooted with comparable effectiveness, from approximately 80 % to 98.5 % (Table 1, analysis in ‘rooting’ column, marked by upper case letters). The only exception—59 %—was observed for line LT11-18C, which, however, multiplied at the highest rate. As a result, only a low or exceptionally a medium correlation (positive or negative) was found between multiplication and rooting for LT lines. However, it was still noticeable in comparison to a lack of correlation for the control lettuce. Micropropagation effectiveness, which was the product of multiplication and rooting, ranged from approximately 3.5 to 5.8. As a result, potential total micropropagation yield would amount to approximately 500–6600 plant clones, depending on the line (respective micropropagation effectiveness raised to the power of the number of multiplication passages—here 5).

**Table 1:**
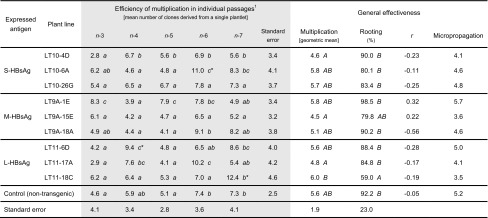
Micropropagation of lettuce T1 plants expressing HBV surface antigens (HBsAg)

The signs in the block of individual micropropagation passage (grey coloured): lowercases in rows mark statistically homogenous groups within passages of a particular line; asterisks in columns mark statistically significant differences between a particular transgenic line and the control within the same passages. Uppercases in columns of general effectiveness mark statistically homogenous groups, separately for multiplication (calculated after logarithmic transformation of geometric mean) and rooting. Statistical analysis made by one-way ANOVA, Tukey HSD test, *p* = 0.01. Correlation coefficient (*r*) refers to the multiplication and rooting. Total micropropagation effectiveness represents the mean number of clones obtained from a single initial plant and was calculated as the product of general multiplication and rooting coefficient

^1^Passages *n*-1 and *n*-2 were conducted without division (multiplication efficiency = 1) on the selective induction medium LM1. Next passages were conducted on the LM2 medium

### HBsAg expression

**Fig. 3:**
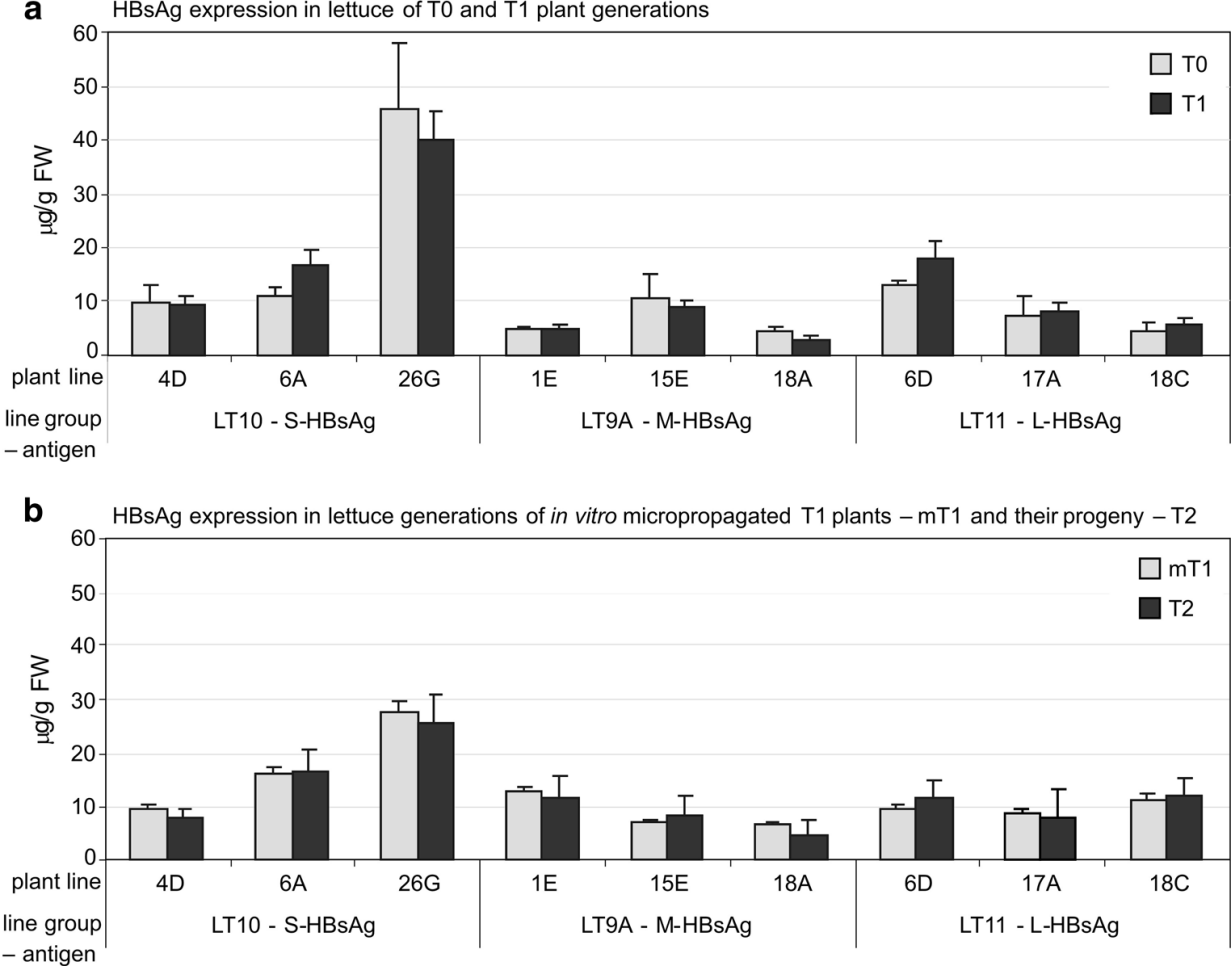
Mean expression level of HBsAg (µg/g FW) in consecutive generations of transgenic lettuce lines. **a** Juxtaposition of primary transformants (T0) and their progeny (T1); **b** comparison of micropropagated T1 transformants (mT1) and their progeny (T2)—for each line, ten plants per each of three randomly chosen plants of the mT1 generation

The lines of transgenic lettuce used for the experiments were selected on the basis of primary test for HBsAg expression in ten plants of the T1 generation, where the expression was similar to that for T0 plants (Fig. 3a). Analysis of HBsAg expression across a large number of micropropagated and reference plants of the selected lines revealed several tendencies (Table 2). Depending on the line, micropropagated plants (clones) growing under ex vitro conditions varied in the expression of particular HBsAg proteins (S-, M- or L-HBsAg), yet there was no clone failing to produce HBsAg. In general, HBsAg content in ex vitro clones reached similar values as in reference plants, but some fluctuations occurred. Interestingly, for lines with the highest HBsAg expression in reference plants (LT10-26G, LT9A-15E and LT11-6D), the largest relative drops in antigen content in their ex vitro clones was recorded—up to ratios of 0.68, 0.74 and 0.54, respectively. An opposite tendency was observed for lines with the lowest HBsAg expression in reference plants (LT9A-1E and -18A and LT11-18C)—with ratios of 2.43, 2.26 and 1.83, respectively. For medium lines (LT10-4D and -6A and LT11-17A) mean HBsAg contents in reference plants and clones did not shift considerably—with change ratios at 1.03, 0.93 and 1.03, respectively. Nevertheless, for almost all lines, the percentages of clones with mean HBsAg values statistically equivalent to the mean for reference plants amounted to around 90 % and above, except for LT9A-1E at 64 %. Biological variability measured by standard errors of mean HBsAg expression in cloned plants reached only 0.3–0.65 of those recorded for the respective reference plants. Coefficients of variation of HBsAg content between clones and reference plants were close—with ratios ranging from 0.7 to 1.5. Yet, considering that the number of clones exceeded four to eight times that of reference plants, clones can be perceived as several times more aligned with regard to HBsAg expression. Uniformisation of HBsAg production in ex vitro plants was further confirmed by fractions of initial plants in variation ranges (<10, 10–20 and >20 %) between clones. For six out of nine lines, most clones (56–85 %) expressed less than 10 % variation in antigen expression. Only up to 10 %, exceptionally 12 % (for line LT10-6A), of clones showed a greater than 20 % difference from the mean expression level for a given line.

**Table 2:** Expression of HBV surface antigens (S-, M-, L-HBsAg) in lettuce plants after micropropagation (ex vitro clones) in comparison to reference plants—constantly growing in standard greenhouse conditions

Expressed antigen	Plant line	Mean value ± SEM of HBsAg expression in reference plants^1^ (µg/g FW)	Coefficient of variation between reference plants	Mean value ± SEM of HBsAg expression in ex vitro clones^2^ (µg/g FW)	Coefficient of variation between clones	Percentage of cloned plants with mean HBsAg values equivalent to the mean for reference plants (*p* < 0.01)	Percentage of initial plants with the coefficient of variation between clones in the range			*p* value for testing the equality of variance between clones of particular plants
							<10 %	10–20 %	>20 %	
S-HBsAg	LT10-4D	9.24 ± 1.83	8.67	9.51 ± 0.74	13.10	94.44	38.24	58.82	2.94	0.037
	LT10-6A	16.58 ± 2.92	10.73	15.44 ± 1.78	15.68	92.31	56.00	32.00	12.00	<0.001
	LT10-26G	40.11 ± 5.12	13.72	27.22 ± 1.97	10.46	100.00	73.91	26.09	0.00	0.027
M-HBsAg	LT9A-1E	5.12 ± 0.74	14.07	12.43 ± 0.84	13.04	63.64	63.64	31.82	4.54	<0.001
	LT9A-15E	8.84 ± 1.15	8.92	6.58 ± 0.53	9.34	92.00	78.26	21.74	0.00	0.040
	LT9A-18A	2.78 ± 0.64	17.19	6.28 ± 0.44	12.02	100.00	84.62	7.69	7.69	<0.001
L-HBsAg	LT11-6D	17.79 ± 3.08	13.45	9.52 ± 0.75	12.92	90.91	46.88	46.87	6.25	0.011
	LT11-17A	8.16 ± 1.41	13.37	8.44 ± 0.78	13.59	96.97	39.39	51.52	9.09	0.025
	LT11-18C	5.97 ± 0.96	13.72	10.91 ± 1.01	9.67	86.87	77.27	18.18	4.55	0.645

The data were analysed statistically (after log_2_(*x*) transformation, for each plant line separately) by analysis of variance in a mixed model involving fixed effects of cloned plants and random effects of clones and allowing for heterogeneous variance between clones

^1^Reference plants—21 to 35 (≥70 %) of PCR—positively verified from 30 to 35 sown

^2^Number of ex vitro clones counted 6-8 per an individual plant (except 4 for LT11-18C), i.e. 22-35 (≥ 70%) of PCR-positively verified from those 30 - 35 developed on the selective induction medium LM1 (passages n-1 and n-2) = 104 - 250 in total.

### Plant growth under ex vitro conditions

Apart from HBsAg expression, the obtained clones were characterised for some basic traits of growth and development—biomass and fertility defined as seed production. Morphologically, mature micropropagated plants did not differ from reference or control non-transgenic plants, constantly growing in the greenhouse (Fig. 2f). Furthermore, for all lines, leaf and total biomass of ex vitro clones was statistically homogenous with their respective reference as well as control non-transgenic plants (Table 3, separate analyses for columns ‘leaves’ and ‘total’). However, some impact of in vitro conditions on subsequent plant development was observed with regard to fertility (Table 3, ‘seed number’ column). The number of seeds produced by individual clones varied considerably—approximately 1.5–2 times more than for reference plants. The mean number of seeds produced by an individual clone constituted 0.64–0.87 of that for a reference plant. Still within a line, the mean number of seeds for clones and their reference plants was statistically uniform (Table 3, analysis marked by lowercase letters, compare relevant pairs in rows for a particular line in ‘seed’ column). Moreover, the means for both plant types corresponded to the respective control plants (on comparing micropropagated lines vs. micropropagated controls and reference lines vs. reference controls, respectively). Despite a decreased seed production by ex vitro grown micropropagated plants of the T1 generation (mT1), progeny plants (T2 generation) of randomly chosen clones developed without aberrations, as well as expressed HBsAg in a pattern similar to that observed in T1 reference plants vs. T0 (Fig. 3).

**Table 3:** Development ex vitro of plants obtained by micropropagation in comparison to reference plants—constantly growing in standard greenhouse conditions

Antigen	Plant line	Development route of plants	Mean^1^ biomass (g)		Mean^2^ number of seeds
			leaves	total	
S-HBsAg	LT10-4D	Micropropagated	34.3 *a*	35.8 *a*	1770 *de*
		Reference	34.1 *a*	35.1 *a*	2334 *e*
	LT10-6A	Micropropagated	31.6 *a*	32.9 *a*	1609 *bde*
		Reference	33.9 *a*	35.3 *a*	2493 *e*
	LT10-26G	Micropropagated	30.6 *a*	31.6 *a*	762 *a*
		Reference	36.8 *a*	38.4 *a*	1070 *abcd*
M-HBsAg	LT9A-1E	Micropropagated	34.2 *a*	35.6 *a*	1060 *abc*
		Reference	44.0 *a*	45.5 *a*	1416 *abcde*
	LT9A-15E	Micropropagated	27.3 *a*	28.5 *a*	1161 *abc*
		Reference	25.2 *a*	26.3 *a*	1690 *bcde*
	LT9A-18A	Micropropagated	36.8 a	37.9 *a*	1076 *abc*
		Reference	39.6 *a*	40.9 *a*	1391 *abcde*
L-HBsAg	LT11-6D	Micropropagated	33.9 *a*	35.1 *a*	807 *a*
		Reference	31.5 *a*	33.0 *a*	925 *abcd*
	LT11-17A	Micropropagated	38.7 *a*	39.7 *a*	943 *ac*
		Reference	29.6 *a*	31.0 *a*	1116 *abcd*
	LT11-18C	Micropropagated	38.0 *a*	39.0 *a*	671 *a*
		Reference	42.5 *a*	43.4 *a*	858 *abc*
Control (non-transgenic)		Micropropagated	30.7 *a*	32.0 *a*	1019 *abcd*
		Reference	33.7 *a*	34.9 *a*	1412 *abcde*
Standard error			8.4	8.5	1051

Letters mark statistically homogenous groups (according to one-way ANOVA, Tukey HSD test, *p* = 0.01) separately for leaf and total biomass and seed number

^1^Mean for t reference plants or 30 micropropagated ones—6 per each of 5 micropropagation passages

^2^Mean for all plants remaining after biomass measurement

## Discussion

Tissue cultures of lettuce are usually used for generation of new forms, including transgenic plants, to induce variation and broaden the genetic base for breeding purposes and sometimes in physiological and biochemical studies (Curtis 2006; Davey et al. 2007; Lebeda et al. 2014). Vegetative micropropagation is rarely applied for lettuce production and, if so, as a complement in the generation of valuable hybrid or inbred lines (Pink and Carter 1987; Pink 1992; Maisonneuve et al. 1995). However, different micropropagation techniques—meristem cultures, organogenesis in callus or somatic embryogenesis, as well as cell and suspension cultures, are used to produce medicinal plants or directly bioactive secondary metabolites (Debnath et al. 2006; Lucchesini and Mensuali-Sodi 2010). Here, we found a new application for micropropagation to produce transgenic lettuce plants containing recombinant proteins of oral vaccine importance.

The aim of the presented research was to develop a micropropagation procedure for transgenic lettuce lines for uniform production of leaf tissue containing particular HBsAg proteins. Thus, apart from the elaboration of an in vitro culture system, these studies required an analysis of the impact of tissue culture period on HBsAg expression and plant growth ex vitro.

In the presented micropropagation method, new plants (clones) were obtained from multiplantlets formed by KIN-stimulated axillary buds of initial seedlings or later from derived plantlets. In comparison to previous attempts in micropropagation, summarised by Pink (1992), we used truncated seedlings as initial explants instead of excised buds, and in place of MS medium (Murashige and Skoog 1962) supplemented with BAP or KIN and IAA or IBA, we applied mixed SH-B5 media (see “Materials and methods”) supplemented only with KIN at various concentrations for multiplication, as well as depleted medium without any growth regulators for rooting. A combination of a suitable explant type and media sequence has resulted in a considerable effectiveness of lettuce micropropagation, several times higher than that reported previously. Truncated seedlings appeared to be more vigorous than isolated meristems, in addition to being more susceptible to the activity of exogenous cytokinin when also deprived of cotyledons (Fig. 1). In turn, the use of media with mild cytokinin—KIN as the only growth regulator, at first at high dose in LM1 medium to induce axillary bud development and then lowered in LM2 medium, resulted in a considerable rate of multiplication of properly developed plantlets. The continuous culture on LM1 medium was not tested (see “Results”), as a prolonged use of high cytokinin concentrations was considered overstimulative in bud formation, which would result in dwarfing of derived plantlets. According to the established protocol, multiplication efficiency did not differ significantly for most lines, including the control lettuce, indicating that both antigen type and its expression level did not affect the development of cultured plantlets (Table 1; Fig. 2). Also, plantlets multiplied reasonably steadily across passages despite fluctuations observed for some lines. Moreover, for most lines, the in vitro culture period did not affect their capability of rooting. For transgenic lines, we noticed only low (exceptionally medium) correlations between multiplication and rooting, in comparison to a lack of correlation for the control lettuce. This may indicate indefinite small pleiotropic effects exerted by inserted transgene copies (see “Materials and methods”). Altogether, the applied micropropagation protocol made it possible to obtain potentially 500–6600 derivative clones from an initial plant within 4–5 months.

Earlier, micropropagation via callus cultures was reported as a method making it possible to achieve many more shoots for artificial seeds (Pink 1992). Nevertheless, we decided to develop a system based on stimulation of organised meristems in pre-existing buds instead of redifferentiation of adventitious buds. The second approach is burdened with a higher probability of spontaneous somaclonal variation in callus and the risk of deleterious alterations in regenerants (Brown et al. 1986; Swartz 1991). Organogenesis also implies an increased complexity and a longer time requirement (Michelmore et al. 1987; Enomoto et al. 1990; Pink 1992; Torres et al. 1993; Pniewski et al. 2011). Although the presented method of in vitro lettuce micropropagation constitutes a self-contained system, it could be further optimised, e.g. in the direction of hydroponic cultures (Kozai 1991; Resh 2013; Su et al. 2015).

Micropropagation made it possible to obtain a large number of plants of uniformised HBsAg expression. In primary tests, we selected lettuce lines of comparable HBsAg content in T0 and T1 plants (Fig. 3a), as we intended to avoid lines of unstable, presumably silenced expression. Therefore, although not being among the best of the T0 generation, the selected lines represented some spectrum of relatively medium and high expression, considering approximately 10–45 µg/g FW, 4–10.5 µg/g FW and 4–13 µg/g FW for S-, M- and L-HBsAg, respectively, in comparison to the previously obtained 0.02–60 µg S-HBsAg/g FW or from nanograms to 16–17 µg M- or L-HBsAg/g FW (Pniewski et al. 2011, 2012). To ensure variability between the lines, some of those lines characterised as having similar HBsAg expression level in the T0 generation were selected on the basis of differing in the number of transgene integration sites (see “Materials and methods” and “Plant material”). The HBsAg expression in the obtained clones was analysed after their transfer to ex vitro conditions (Tables 2, 3). Most importantly, the content of particular HBsAg proteins in micropropagated and reference plants was comparable (see Table 2 and “Results”) showing no impact of in vitro culture on HBsAg expression. Although the specific conditions and processes occurring during in vitro cultures may alter gene expression (Brown et al. 1986; Swartz 1991), this effect was not observed here, probably due to micropropagation via stimulation of axillary buds instead of organogenesis de novo. In parallel, the clones were characterised as having a high degree of uniformisation regarding HBsAg expression, as variation among clones was several times lower than between the respective reference plants, growing without the impact of tissue culture. The observed deviations may even indicate a tendency that micropropagation affected, to some extent, equalisation of HBsAg expression between lines, i.e. promoted its increase for low-expressing lines and acted inversely for high-producing ones. HBsAg production was especially equalised between clones obtained from an individual plant. Practically for clones of all tested plants, variation coefficients of HBsAg content did not exceed 20 % and were below 10 % for approximately 40–85 % of clones. The observed fluctuations of S-, M- and L-HBsAg expression correspond to data obtained for the production of various medicinal substances, including low-weight compounds, in standardised plant cell cultures or similar systems (Kim et al. 2004; Ghasemzadeh et al. 2016). Hence, such a degree of variation of HBsAg expression could be accepted for possible production of plant material for an anti-HBV vaccine. Plant biomass, as equally important for manufacturing as stabilised HBsAg expression, also did not fluctuate significantly among transgenic lines, both for micropropagated and reference plants, as well as in comparison to non-transgenic control (Table 3). The only adverse impact of in vitro culture was observed in terms of decreased seed production; yet, this reduction was still statistically insignificant for a line (see Table 3 and Results). However, due to the multiplied number of clones, the total seed production was even four to seven times larger than for reference plants. Observations of ten plants of the T2 generation, progeny of three randomly chosen micropropagated T1 plants (mT1) of each tested line, showed no aberration in growth. HBsAg expression was comparable between T2 plants and the respective mT1—here parental plants, similarly to that for T0 and T1 plants (Fig. 3). Thus, micropropagation could even be considered as a method to increase the scale of seed production for the continuous process of fabrication of plant material bearing antigens.

Altogether, the presented micropropagation protocol makes it possible to substantially increase the production scale of lettuce of relatively high and considerably uniformised contents of S-, M- and L-HBsAg. This, together with the reported optimisation of plant tissue conversion to lyophilisate and its long-term stability (Czyż et al. 2014, 2016), makes it a successive essential step in manufacturing of a standardised oral vaccine against HBV of reliable efficacy. As lettuce becomes more and more attractive as a platform for expression of various heterologous proteins (Dong et al. 2014), our results show that micropropagation can serve as a biofarming tool, enabling a considerable increase in the yield of production of biopharmaceuticals.

### **Author contribution statement**

TP and JK conceived and designed the study. TP, MC and KW executed in vitro cultures and plant analyses. PB performed the pilot experiment. PK, MC and TP performed statistical analysis. TP wrote the paper.

## Abbreviations

BAP: Benzylaminopurine
IAA: Indolylacetic acid
IBA: Indolylbutyric acid
KIN: Kinetin
HBV: Hepatitis B virus
HBsAg: Hepatitis B surface antigen
SEM: Standard error of the mean
